# Survival and gene expression under different temperature and humidity regimes in ants

**DOI:** 10.1371/journal.pone.0181137

**Published:** 2017-07-31

**Authors:** Dimitri Stucki, Dalial Freitak, Liselotte Sundström

**Affiliations:** 1 Centre of Excellence in Biological Interactions / Department of Biosciences, University of Helsinki, Helsinki, Finland; 2 Tvärminne Zoological Station, University of Helsinki, Hanko, Finland; 3 Centre of Excellence in Biological Interactions, University of Jyväskylä, Jyväskylä, Finland; Fred Hutchinson Cancer Research Center, UNITED STATES

## Abstract

Short term variation in environmental conditions requires individuals to adapt via changes in behavior and/or physiology. In particular variation in temperature and humidity are common, and the physiological adaptation to changes in temperature and humidity often involves alterations in gene expression, in particular that of heat-shock proteins. However, not only traits involved in the resistance to environmental stresses, but also other traits, such as immune defenses, may be influenced indirectly by changes in temperature and humidity. Here we investigated the response of the ant *F*. *exsecta* to two temperature regimes (20°C & 25°C), and two humidity regimes (50% & 75%), for two populations. We measured the survival and the expression of six metabolism- and immunity-related genes, and furthermore compared the expression levels in each condition with the pre-experiment expression levels. Both populations survived equally well at the two humidities, but one population showed higher mortality at 25°C than 20°, at 50% humidity. Similarly, the two populations showed striking differences in their gene expression before the experiment, and in their responses to the environmental conditions. Surprisingly, instead of converging to similar expression levels in the same environmental conditions, gene expression diverged further apart. This indicates different reaction norms to both temperature and humidity for the two populations. Furthermore, our results suggest that also immune defenses are indirectly affected by environmental conditions.

## Introduction

Individuals respond to short term changes in environmental conditions, either via altering behavior or via adjusting physiological processes mediated by modifications in gene expression patterns [1]. These responses are often not identical among individuals within a species, although the conditions may be similar, but are determined by reaction norms specific to e.g. populations or individuals [2]. These reaction norms depend on the genetic makeup of an individual, but are also determined during development [3], or via transgenerational effects [4]. Furthermore, short-term acclimatization to different temperatures can result in differential expression of heat-shock genes [5]. The resistance of an individual to environmental stress, such as heat- and cold-tolerance, or resistance to desiccation, is often sculpted by the conditions met during development [6,7]. In particular, temperature and moisture can shape reaction norms, as they often vary in space and time. Such variation in temperature, or moisture, can result in a differential expression of genes directly related to coping with changes in these conditions, such as the expression of heat-shock proteins [8]. Also other genes not directly linked to temperature or moisture, such as immune defenses, can show changes in expression patterns [9–11]. For example, *Drosophila* flies from different populations showed reduced immune defenses at temperatures that differed from their naturally encountered temperature [11]. This indicates that the expression of immune defense traits may depend on the reaction norm to the experienced conditions.

The most frequent physiological response to variation in temperatures and humidity is by altering the expression of heat- and cold-shock proteins [12–15]. Also other genetic pathways have been linked to environment-dependent processes, such as the insulin-like protein pathway in overwintering insects [16], and metabolism-related genes under drought [17]. For example, immune genes from the Toll and PPO pathways are differentially expressed under different temperature regimes in the alfalfa leafcutting bee *Megachile rotundata* [18], and and metabolism-related genes were generally down regulated during desiccation in the Antarctic mite, *Belgica Antarctica* [17]. Similarly, a recent study on two ant species showed a high amount of genes to be temperature-sensitive, including potential immune genes [19].

Given that temperature and humidity often affect the development of fungal pathogens [20–22], a reaction norm that raises immune defenses at higher temperatures and/or humidities may thus increase the fitness of an individual, but may also incur a fitness cost in the absence of an infection. Therefore, although environmental conditions can have a direct influence on the expression of immune traits, the precise nature of this influence depends on the reaction norms of an individual. Given such complex interactions, it may be difficult to predict how individuals regulate immune defenses under different environmental conditions. Nevertheless, this information is important for understanding host-parasite interactions in the light of eco-evolutionary processes under variable environmental conditions.

Here we investigated how workers of two natural populations, one island population and on mainland population, of the ant *F*. *exsecta* respond to different environmental conditions in a controlled laboratory experiment. We transferred field-collected ants from the two populations to the laboratory, maintained them at four temperature-humidity combinations, and measured the change in the expression of six immunity- and metabolism-related genes. We selected a heat-shock protein (*HSP75*), an insulin receptor (*IR3*), a hemolymph storage protein, which however also shows immune functions (Arylphorin), and three immune genes (β-1,3-glucan-binding protein, Pro-Phenoloxidase, Toll-receptor). In addition, we recorded the survival of the ants under each environmental condition. The colonies of the island population are mostly located on dry soil and exposed to the sun, whereas the colonies of the mainland population are located in a forest with closed canopy, and a rich vegetation. Thus, we expected the ants of the two colonies to respond differently to the conditions, in particular, we expected a stronger response to the conditions the colonies are less accustomed to. Furthermore, we expected gene expression to be elevated in low temperatures, as well as in the dry conditions.

## Material & methods

### Sampling & bioassay

We collected *Formica exsecta* workers from inside the nest, together with ~200ml nest material, between the 20^th^ and 23^rd^ of June 2014. In total we collected 1932 worker ants from 21 colonies (92 ants per colony), twelve of which originated from an island population (Furuskär: *59°50'00"N 23°16'05"E*), and nine from a mainland population (Prästkulla: *59°58'45"N 23°20'51"E*). We henceforth refer to these populations as island population (Furuskär) and mainland population (Prästkulla). From each colony, we immediately sampled four biological replicates to measure gene expression before starting the experiment. Each biological replicate consisted of three ants, which we fixed (directly cut into small pieces) in 300μl Isol-RNA Lysis Reagent (5 Prime), and stored them at -80°C until further processing. With the remaining ants from the respective colonies, we established the laboratory treatments. From each colony we placed 20 workers into each of four Fluon® coated pots (Ø: 7cm, h: 5cm) with plaster lining (i.e. four pots per colony). Each pot was then randomly assigned to one of two temperature-treatments (20°C and 25°C) and to one of two humidity-treatments (50% and 75%) in a full factorial design. The pots were placed in incubators set to the respective temperature and 50% relative air humidity. We also added an open 1.5ml tube filled with water and a piece of cotton to each pot of the 75% humidity-treatment, and confirmed that the humidity indeed increased and was maintained at ca. 75%. The pots in the 50% humidity treatment remained without a water tube. For simplicity, we refer to the conditions as cold/hot (20°C / 25°C) and dry/moist (50% / 75%). We then fed the ants daily with ~200μl Bhatkar-Whitcomb diet [23], and removed and recorded dead individuals from the pots daily. On day ten after the onset of the laboratory treatments, we sampled three biological replicates from each pot for gene expression analysis to determine the effect of the laboratory conditions. For each biological sample, we fixed three ants (directly cut into small pieces) into 300μl Isol-RNA Lysis Reagent, and stored them at -80°C until further processing.

### Gene expression analysis

We first homogenized the thawed samples in a total volume of 600μl RNA Isol-RNA Lysis Reagent, with two stainless steel beads using a TissueLyser (Qiagen). Subsequently we added 400μl Isol-RNA Lysis Reagent and 150μl 1-Bromo-5-Chloropentane (Sigma). After mixing we centrifuged the samples for 10min at 13,000rpm at 4°C. We then transferred the upper, transparent phase, containing the RNA, to a new 1.5ml tube and supplemented with 500μl isopropanol (Sigma). After mixing, we let the suspended RNA precipitate over night at -20°C, and then centrifuged the samples for 30min at 13,000rpm at 4°C to sediment the RNA. After removal of the supernatant, we washed the pellet on ice with 500μl 80% EtOH (Altia Oyj) and centrifuged for 10min at 13,000rpm at 4°C. After drying the pellet we dissolved the RNA in RNA storage solution (AMBION). We measured concentration and quality of the RNA photospectrometrically with a NanoDrop (PEQ-Lab) and eliminated possible DNA contamination by DNAse digest (TURBO DNase; AMBION) before cDNA synthesis (iScript cDNA Synthesis Kit; Bio-Rad). For cDNA synthesis we used 1μg RNA for each sample and afterwards diluted the resulting 20μl cDNA in 80μl autoclaved ddH_2_O.

As target genes we chose six genes, five of which were previously shown to be altered upon infection in workers of this species (unpublished data). We chose three immune genes coding for the antifungal peptide β-1,3-glucan-binding protein (*B1*,*3g*), and two cascade molecules, Pro-Phenoloxidase (*PPO*) and Toll-receptor (*Toll*). We also included three metabolism-related genes coding for the storage protein Arylphorin (*Aryl*), the insulin receptor 3 (*IR3*), and the heat shock protein 75 (*HSP75*). However, both Arylphorin and the insulin pathway have also been shown to be linked to immune responses [24,25]. A list of the primer sequences is provided in the supplementary table A in S1 File.

We designed qRT-PCR primers using the online Primer3 internet-based interface (http://www.ncbi.nlm.nih.gov/tools/primer-blast/) [26]. Primers were designed by the rules of highest maximum efficiency and sensitivity to avoid formation of self- and hetero-dimers, hairpins and self-complementarity. Gene-specific primers were designed on the basis of the sequences obtained from the *F*. *exsecta* transcriptome [27]. Q-RT-PCR was performed on 384-well plates on a CFX384 Touch™ Real Time PCR Detection System (Biorad) using iQ™ SYBR® Green Supermix (Bio-Rad), with a 3-min initiation phase at 95°C, followed by 40 cycles of 15sec at 95°C for denaturation, and 45sec at 58°C for annealing/extension, and a final step of 7min at 95°C. All Q-RT-PCR assays were run using two technical replicates, and assessed for consistency and possible outliers, and subsequently averaged before normalization. Non-detects (no amplification signal within 40 qPCR cycles) were set to the maximal cycle number (i.e. Ct = 40), or removed if the second technical replicate showed amplification.

### Statistical analysis

We used a mixed effects Cox proportional-hazard model to compare the survival across the environmental conditions, as well as between the populations. We specified temperature, humidity, and population as fixed factors, and included all possible interactions. Colony of origin was added as random factor. Subsequently we ran post-hoc contrasts on all pairwise comparisons, which were adjusted for false discovery rates [28]. We then tested for differences in gene expression with Principal Component Analyses on the inverted normalized Ct-values. We inverted the Ct-values because they are negatively correlated with specific transcript level (i.e. higher Ct-values indicate lower gene expression levels). To normalize gene expression we used the NORMA-Gene algorithm, which does not require reference genes for normalization [29]. As suggested by the authors, we nevertheless included the reference gene *RPS9* to stabilize the normalization, but not as a gene of interest. Hence we do not report the results for this gene.

We used two PCAs, one to test for differences in pre-experiment gene expression between the populations, and a second to test for differences in gene expression across the experimental conditions and between the populations. We used unrotated PCAs for component selection and retained all components with an Eigenvalue > 1.0 (Table B in S1 File). We also consulted the scree plot of the Eigenvalues for consistency with the suggested number of components to retain. For the PCA on pre-experiment gene expression the scree plot indicated a high relative weight of a component despite an Eigenvalue < 1.0, and in this case we also included this component. We then rotated the scores of the retained components using oblique (oblimin) rotation. The rotated scores were then used as dependent variables in two linear mixed effects models for each retained cluster (PC), with the PC score as dependent variable. In the pre-experiment analysis we entered Population as a fixed factor, and Colony as a random effect, whereas in the second analysis across the experimental conditions and between the populations we entered the Population, Temperature and Humidity as a fixed factors, and Colony as a random effect. We then performed planned post-hoc comparisons, where we compared for each PC the pairwise differences between the two populations (separately for each experimental condition), and each population the pairwise differences between temperatures (at each humidity) and humidities (at each temperature). We then repeated the same analyses separately for each gene, in order to assess the congruence between the genes highlighted by the PCA, and the specific genes showing differential expression between the population, and treatments. We here arbitrarily define the association of each gene with a principal component as strong (loading 0.67–1.0), moderate (loading 0.33–0.66), or weak (0.0–0.32).

Finally, we compared the pre-experimental status of gene expression with that after ten days of exposure to the laboratory conditions, in linear mixed effects models for each gene separately. We specified the factors as above, and included all possible interactions. To test for pairwise differences, we ran planned pairwise contrasts on the pre-experimental status against the state of gene expression in each environmental condition, and adjusted for false discovery rates as above.

Four samples did not yield sufficient RNA for analysis and were thus removed. For the statistical analyses we used R 3.2.3 [30], and the packages survival [31], coxme [32], lme4 [33], lmerTest [34], and multcomp [35]. For all statistical tests we used a significance threshold of α = 0.05, and correction for false discovery rates where applicable. Variation is indicated as standard errors, unless specified otherwise.

## Results

**Fig 1:**
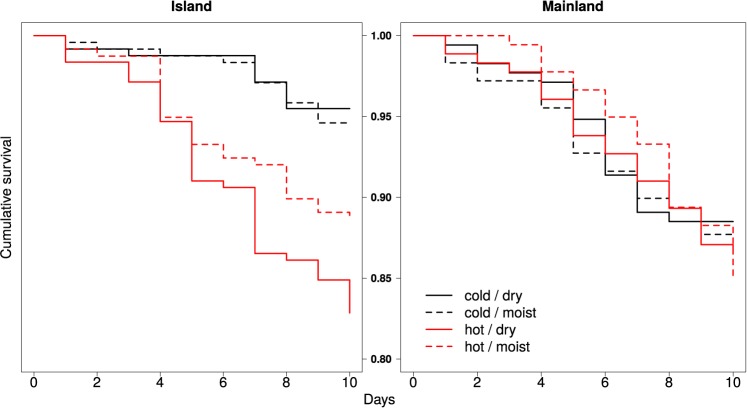
Condition-dependent survival. Cumulative survival during the laboratory experiment for each laboratory condition, separated by population. Solid lines indicate the survival in dry conditions, and dashed lines indicate the survival in moist conditions. Survival at cold temperatures is shown in black, and survival at hot temperatures is shown in red. Mind that the y-axis ranges only between 0.8 and 1.0.

Overall, survival did not differ significantly between the two populations, but temperature had a significant effect on the survival of the ants in one of the two populations, whereas humidity had no effect on survival (Table 1). In particular, ants from the island population died at a significantly faster rate at hot/dry compared to cold/dry conditions, whereas survival did not differ significantly between hot/moist and cold/moist conditions, despite a similar trend (Table B in S1 File, Fig 1). The survival of ants from the mainland population did not differ between the two temperatures, at either of the two humidity levels (Table B in S1 File, Fig 1).

**Table 1:** Survival regression models.

Factor	Chi^2^	p-value
Temp	89.5609	<0.0001
Hum	0.3139	0.58
Pop	0.8167	0.37
Temp:Hum	1.5391	0.22
Temp:Pop	11.5649	0.0007
Hum:Pop	1.4893	0.22
Temp:Hum:Pop	0.7441	0.39

Results of the Cox proportional-hazard regression on survival. Temp indicates the effect of temperature (cold vs hot), Hum the effect of humidity (dry vs moist) and Pop the populations (Island vs Mainland).

**Fig 2:**
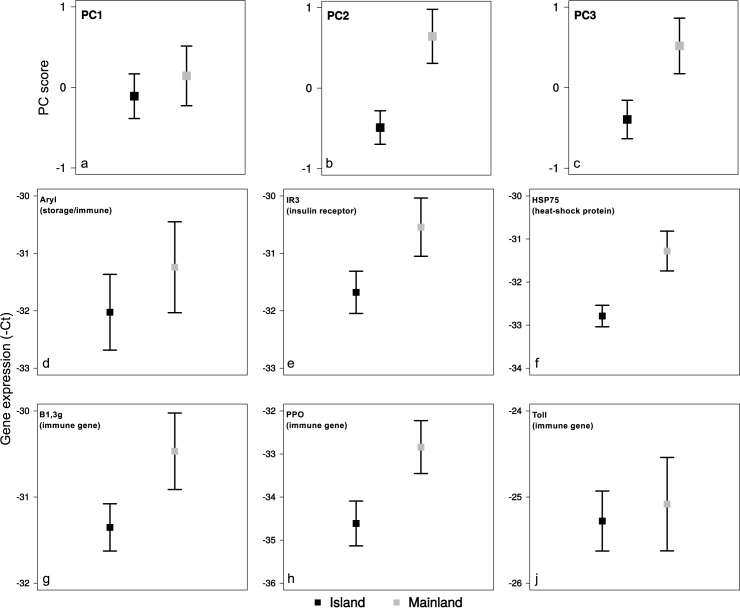
Pre-experiment population differences in gene expression. Population-differences in PC scores (panels a-c) representing pre-experiment gene expression of the six genes (panels d-i). Error bars indicate 95% confidence intervals.

In the PCA we ran on pre-experiment gene expression, two components had an eigenvalue above 1.0 and explained 68% of the variation (Table C in S1 File). However, the scree plot (Fig 1A) suggested to include also PC3 for the analysis, which raised the variation explained to 84%. PC1 explained 47% of the variation, but was not significantly associated with population differences (β±SE = 0.25±0.32, t = 0.77, p = 0.45), whereas both PC2 (21% of variation explained), and PC3 (16% of variation explained) indicated significant differences in gene expression between the populations, with higher PC scores (i.e. gene expression) in ants from the mainland population than the island population (PC2: β±SE = 1.13±0.20, t = 5.75, p<0.0001; PC3: β±SE = 0.91±0.25, t = 3.65, p = 0.0025). The loadings of each PC partly reflected the gene functions. PC1 showed a strong positive association with the two immune genes *B1*,*3g* and *Toll*, and a strong negative association with the gene coding for the storage/immune protein *Aryl*, PC2 showed a strong positive association with the gene coding for the heat-shock protein *HSP75* and the insulin receptor gene *IR3*, and PC3 showed a strong, respectively moderate, positive association with the two immune genes *PPO* and *B1*,*3g* (Table D in S1 File). This suggests that four of the six genes (*HSP75*, *IR3*, *PPO*, *B1*,*3g*) are more expressed in ants from the mainland population than the island population, which the gene-by-gene also fully supports (*B1*,*3g*: β±SE = -0.88±0.31, t = -2.84, p = 0.0158; *HSP75*: β±SE = -1.51±0.26, t = -5.71, p = 0.0001; *IR3*: β±SE = -1.13±0.31, t = -3.70, p = 0.0043; *PPO*: β±SE = -1.76±0.50, t = -3.53, p = 0.0043; Fig 2D–2I). The remaining two genes (*Aryl* & *Toll*) did not show significant differences in expression between the populations, despite the strong negative association of *Aryl* with PC1 (*Aryl*: β±SE = -0.80±0.73, t = -1.10, p = 0.34; *Toll*: β±SE = -0.21±0.46, t = -0.46, p = 0.65; Fig 2D–2I).

**Fig 3:**
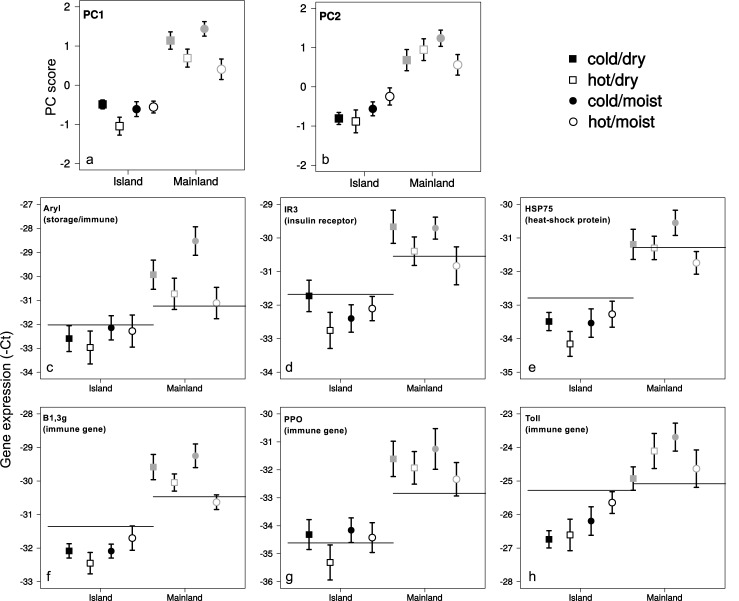
Condition-dependent gene expression levels. PC scores (panels a & b) representing gene expression of six genes (panels c-h) after ten days acclimatization to one of four laboratory conditions. Squares indicate gene expression at dry conditions, and circles indicate the gene expression at humid conditions. Filled symbols represent gene expression at cold temperatures, and open symbols represent gene expression at hot temperatures. Error bars indicate 95% confidence intervals.

The PCA for the experimental conditions produced two components with an eigenvalue above 1.0, which explained 69% of the variation (Table C in S1 File). The scree plot also suggested two components to be sufficient (Fig A in S1B File). Both PCs separated between populations, with higher scores for the mainland population than the island population (Fig 3A & 3B, Tables 2 & E in S1 File), again indicating higher overall expression levels in the mainland population. The loadings on these two PCs captured all the genes under investigation, with PC1 capturing the heat-shock protein *HSP75*, the insulin receptor *IR3*, the storage/immune gene *Aryl*, and the two immune genes *B1*,*3g* and *PPO* (strong: *Aryl*, *HSP75* & *IR3*; moderate: *B1*,*3g* & *PPO*), and PC2 capturing the three immune genes (strong: *B1*,*3g* & *Toll*, moderate: *PPO*; Table D in S1 File). The high and consistently unidirectional (positive) factor loadings suggest that the expression levels, and apparent collinearity among the genes, were strongly influenced by the differences between the populations. In the gene-by-gene analysis the same (six) genes *Aryl*, *B1*,*3g*, *HSP75*, *IR3*, *PPO*, *Toll* were significantly differently expressed between the two populations (Fig 3C–3H, Tables 2 & E in S1 File).

**Table 2:** Effect of experimental conditions on gene expression (PC scores).

	PC1			PC2		
	SS	F-value	adj. p	SS	F-value	adj. p
Temp	14.97	F_1,220.66_ = 57.91	<0.0001	0.09	F_1,220.72_ = 0.26	0.61
Hum	0.54	F_1,220.51_ = 2.10	0.15	4.14	F_1,220.59_ = 11.77	0.0014
Pop	65.47	F_1,19.04_ = 253.30	<0.0001	45.81	F_1,19.21_ = 130.10	<0.000
Temp:Hum	0.00	F_1,220.66_ = 0.00	0.97	1.10	F_1,220.72_ = 3.11	0.16
Pop:Temp	3.71	F_1,220.66_ = 14.36	0.0004	1.50	F_1,220.72_ = 4.27	0.0400
Pop:Hum	0.46	F_1,220.51_ = 1.80	0.18	1.88	F_1,220.59_ = 5.35	0.0433
Pop:Temp:Hum	5.47	F_1,220.66_ = 21.15	<0.0001	6.49	F_1,220.72_ = 18.44	<0.0001

Results from linear mixed effects models on the selected principal components capturing gene expression after ten days at the experimental conditions. Temp indicates the effect of temperature (cold vs hot), Hum the effect of humidity (dry vs moist) and Pop the populations (Island vs Mainland). All p-values were adjusted for false discovery rates.

Apart from capturing overall population differences PC1 predominantly separated between temperatures, whereas PC2 mainly captured effects of humidity on gene expression (Table 2). Both PCs, however, also brought out differences between the populations in how they responded to the combined effects of humidity and temperature, as suggested by the significant interaction terms in the analysis. The PC1 scores for the island population were lower at hot/dry than cold/dry conditions, but not different between hot/moist and cold/moist conditions, whereas the scores for the mainland population were lower at both hot/dry and hot/moist conditions than at cold/dry and cold/moist conditions, respectively (Fig 3A, Table E in S1 File). This suggests that the expression of the genes associated with PC1 (i.e. *Aryl*, *B1*,*3g*, *HSP75*, *IR3*, and *PPO*) was in general higher at cold than hot temperatures. Also within each temperature regime, humidity variation produced different responses: the PC1 scores for the island population did not differ between the cold/dry and cold/moist conditions, but at the scores were higher at hot/moist than hot/dry conditions. The pattern was reversed in the mainland population, for which the scores were higher at cold/dry than cold/moist conditions, as well as at hot/dry than hot/moist conditions (Fig 3A, Table E in S1 File). Thus, overall, gene expression (PC1) appeared to be higher at dry conditions in the island population, and higher at moist conditions in the mainland population.

The PC2 scores did not differ between cold/dry and cold/moist conditions, or between hot/dry and hot/moist conditions for the island population. For the mainland population the PC2 scores were significantly lower at hot/moist than cold/moist conditions, but did not differ between hot/dry and cold/dry conditions. Within each temperature regime, neither of the populations showed a difference between cold/dry and cold/moist conditions, whereas both populations responded, but in opposite directions in the hot temperature treatment, so that the PC2 scores were higher in hot/moist than hot/dry conditions for the island population and lower in hot/moist than hot/dry conditions for the mainland population (Fig 3B; Table E in S1 File).

The pattern shown by the gene-by-gene analysis mirrored the PCA, except that some of the genes with high loadings on the PCs did not show a significant effect for the treatment in the island population, although the direction of change was similar. Thus, in the island population within each humidity regime the gene coding for the heat-shock protein *HSP75*, the insulin receptor gene *IR3* and the immune gene *PPO* showed a significantly higher expression at cold/dry than hot/dry conditions, whereas no significant differences were found for the other genes, or between cold/moist and hot/moist conditions (Fig 3C–3H, Tables 3 & F in S1 File). In the mainland population all genes showed significantly higher expression at cold/moist than hot/moist conditions, whereas no differences were found between cold/dry and hot/dry conditions, although the direction of the change was in the same direction for all genes associated with PC1 (Fig 3C–3H, Tables 3 & F in S1 File). Within each temperature regime all genes were significantly more expressed at hot/moist than hot/dry conditions, whereas no differences were found between cold/moist and cold/dry conditions in the island population (Fig 3C–3H, Tables 3 & F in S1 File). In the mainland population the storage/immune protein coding gene *Aryl*, the gene coding for the heat-shock protein *HSP75* and the immune gene *Toll*) were more expressed at cold/moist than cold/dry conditions, whereas no differences between hot/moist and hot/dry conditions were found (Fig 3C–3H, Tables 3 & F in S1 File).

**Table 3:** Effect of experimental conditions on gene expression.

	*Aryl* (storage/immune gene)			*IR3* (insulin receptor)			*HSP75* (heat-shock protein)		
	SS	F-value	adj. p	SS	F-value	adj. p	SS	F-value	adj. p
Pop	83.35	F_1, 19.02_ = 36.65	<0.0001	148.56	F1, 19.17 = 101.24	<0.0001	148	F1, 19.25 = 155.65	<0.0001
Temp	58.62	F_1, 220.38_ = 25.77	<0.0001	25.5	F1, 220.95 = 17.38	0.0001	10.79	F1, 220.85 = 11.35	0.0012
Hum	17.14	F_1, 220.29_ = 7.54	0.0197	0.94	F1, 220.76 = 0.64	0.43	4.16	F1, 220.7 = 4.37	0.0753
Pop:Temp	33.51	F_1, 220.38_ = 14.73	0.0005	4.76	F1, 220.95 = 3.24	0.12	2.89	F1, 220.85 = 3.04	0.12
Pop:Hum	0.12	F_1, 220.29_ = 0.05	0.82	0.73	F1, 220.76 = 0.5	0.58	1.41	F1, 220.7 = 1.49	0.336
Temp:Hum	9.8	F_1, 220.38_ = 4.31	0.12	3.07	F1, 220.95 = 2.09	0.30	0.08	F1, 220.85 = 0.09	0.92
Pop:Temp:Hum	17.13	F_1, 220.38_ = 7.53	0.0079	11.18	F1, 220.95 = 7.62	0.0079	15.04	F1, 220.85 = 15.82	0.0003
	*B1*,*3g* (immune gene)			*PPO* (immune gene)			*Toll* (immune gene)		
	SS	F-value	adj. p	SS	F-value	adj. p	SS	F-value	adj. p
Pop	100.36	F1, 19.24 = 177.96	<0.0001	326.73	F1, 19.21 = 134.73	<0.0001	126.78	F1, 19.36 = 105.71	<0.0001
Temp	12.3	F1, 220.74 = 21.81	<0.0001	26.92	F1, 221.17 = 11.1	0.0012	1.24	F1, 221.12 = 1.04	0.31
Hum	0.88	F1, 220.61 = 1.57	0.26	3.7	F1, 220.94 = 1.53	0.26	18.37	F1, 220.93 = 15.32	0.0007
Pop:Temp	12.81	F1, 220.74 = 22.71	0	0.1	F1, 221.17 = 0.04	0.84	2.3	F1, 221.12 = 1.92	0.20
Pop:Hum	3.69	F1, 220.61 = 6.55	0.0669	4.57	F1, 220.94 = 1.88	0.34	2.41	F1, 220.93 = 2.01	0.34
Temp:Hum	0.09	F1, 220.74 = 0.17	0.92	0.01	F1, 221.17 = 0	0.96	6.64	F1, 221.12 = 5.54	0.12
Pop:Temp:Hum	10.33	F1, 220.74 = 18.32	0.0002	8.67	F1, 221.17 = 3.58	0.0599	17.55	F1, 221.12 = 14.63	0.0003

Results from linear mixed effects models on the expression of each gene after ten days at the experimental conditions. Temp indicates the effect of temperature (cold vs hot), Hum the effect of humidity (dry vs moist) and Pop the populations (Island vs Mainland). All p-values were adjusted for false discovery rates.

We next compared the gene-by-gene expression levels between the pre-experimental (control) group and the ants exposed to different temperature and humidity regimes. After ten days in laboratory conditions, the ants from the island population showed reduced expression of the genes *B1*,*3g*, *HSP75* and *Toll*, at cold/dry and cold/moist conditions, and additionally reduced expression of *IR3* at cold/moist conditions (Fig 3C–3H, Table G in S1 File). The ants kept at hot/dry conditions showed reduced expression of all investigated genes, whereas at hot/moist conditions none of the genes were expressed differentially compared to the pre-experimental status (Fig 3C–3H, Table G in S1 File). The ants from the mainland population showed the opposite pattern when compared to the pre-experimental samples. When kept at cold/dry conditions, expression of the storage/immune protein coding gene *Aryl*, the insulin receptor gene *IR3*, and the two immune genes *B1*,*3g* and *PPO* increased, and at cold/moist conditions all genes were up-regulated (Fig 3C–3H, Table G in S1 File). At hot/dry conditions only the immune gene *Toll* showed increased expression, and at hot/moist conditions none of the genes showed a significantly change in gene expression in comparison to the pre-experimental status (Fig 3C–3H, Table G in S1 File).

## Discussion

Our results revealed substantial differences between the populations, with respect to both their ability to cope with different temperature conditions, and in their gene expression patterns under the different environmental regimes. Survival was not directly influenced by humidity, yet, humidity influenced the susceptibility to temperature conditions in one of the two populations. This indicates differences in how the ants from the two populations respond to variation in temperature as well as humidity. Such differences were also reflected in the different patterns of gene expression among the environmental regimes, as well as between the onset and the end of the experiment. In general, gene expression was higher in the mainland population, both at the beginning of the experiment, and especially after exposure to the environmental regimes. This was due to opposite responses to the experimental conditions compared to the pre-experiment level of gene expression. Taken together, these results suggest considerable variation among populations in the responses to physical environmental conditions, with repercussions for both survival and gene expression patterns.

Temperature drastically influenced survival in the island population, with low survival under hot and dry conditions. No effect of temperature on survival was found for the mainland population. In terms of gene expression, transcript levels were overall lower under cold than hot conditions. However, the island population showed a humidity-dependency, and showed this effect only under dry conditions. This supports our expectations that expression levels are higher under cold conditions and agrees with previous studies that found increased gene expression in colder environments in ectotherms [36,37], including ants [19]. This may act to compensate for a generally reduced metabolic rate [38,39]. Alternatively, the reduced gene expression at the higher temperature may reflect desiccation-stress, and potential desiccation-resistance by limitation of the water consumed in protein synthesis [17]. This is supported by the observation that ants from the island population had a significantly higher mortality at hot than cold conditions, but only in the dry environment. Yet, no such differential survival was found for the mainland population, which may reflect variation in the susceptibility to changes in environmental conditions.

Both populations survived equally well at the two humidities, when allowing for the effects of temperature on the island population. The patterns of gene expression in response to the two levels of humidity differed, however, between the populations. In general, ants of the island population showed higher transcript levels in moist than and dry conditions (but only at the higher temperature), whereas, the mainland population predominantly showed higher transcript levels in dry than moist conditions (but only at the lower temperature). This supports our expectation that the responses to the conditions, may depend on the environmental conditions that the ants of the two populations have experienced in their habitat. Humidity has been mostly investigated in the light of desiccation resistance [17,40,41], but its effects on metabolism- and immune gene expression has rarely been studied. The differential expression of the heat-shock protein *HSP75* and the insulin receptor *IR3* likely reflects a role of metabolism-related genes in desiccation resistance [17]. In contrast, the involvement of the immune gene *PPO* suggests a direct influence of environmental humidity on the expression of genes involved in immune defenses. This may relate to the observation that desiccation resistance can be traded off against immune functions [42], and is further supported by the observation that gene expression was generally lower in the dry conditions.

Our results indicate population-dependent responses to temperature and humidity, which may reflect the differences in the natural habitat of these two populations. The colonies of the island population are mostly located on dry soil and exposed to the sun, whereas the mainland population is located in a forest with closed canopy, and a rich vegetation. Thus, the colonies likely experience different natural temperatures and humidity in their habitat. The two populations also differed in their pre-experiment level of gene expression, which likely reflects adaptation (or acclimatization) to the habitat. The generally higher gene expression in the mainland population may, thus, relate to the likely lower temperatures in the forest [36,37]. Nevertheless, when kept at the same experimental conditions, the levels of gene expression did not converge, but instead diverged, leading to even larger population differences. This suggests that the level of gene expression was not only specified by the respective conditions, but also by the specific reaction norms of the individuals. This is also supported by the observation that the conditions which induced the strongest change (in terms of the number of genes differentially regulated) differed for the two populations. In the island population all genes were down-regulated under hot and dry conditions, whereas in the mainland population all genes were up-regulated under cold and moist conditions. Thus, contrary to expectations, the ants responded to the conditions that represented the conditions they likely meet in their habitat. Only at hot and moist conditions did neither of the populations show changes in gene expression compared to the pre-experiment level. This may represent conditions close to those experienced in the nest (but not necessarily the habitat) [43,44]. This suggests variation in the reaction norms between the populations, specific to their natural habitat.

The differences between the two populations may also reflect differences in the social structure of the colonies. The ants of the island population originated from monogyne colonies (headed by a single queen) [45], whereas the colonies in the mainland population were all polygyne (headed by multiple queens). Because polygyne colonies exhibit a higher genetic variation than monogyne colonies [46], it is possible that this provided better resilience against the stressful conditions in the laboratory for the ants in the mainland population, but not for the ants in the island population. Indeed, the ants from the mainland population colonies did not show differences in survival among the laboratory conditions. Thus the diversity of the genetic background may influence the magnitude of response available across the different individuals within each colony, and allow polygyne colonies to more flexibly respond to environmental changes.

Here we have shown that temperature and humidity have a direct influence on the expression level of metabolism- and immunity-related genes in worker ants. This influence may relate to the metabolic activity and/or desiccation resistance of the ants, but was also dependent on the population of origin. This indicates that the individuals from the two populations exhibit different reaction norms in their response to environmental conditions. These reaction norms also involved genes that are not directly associated to the resistance to temperature- and desiccation-stress, like immune genes. As a consequence, changes in environmental conditions may not only lead to an adaptation to the respective conditions, but may also generate variation in other traits, such as immune defenses. The effect of temperature on the gene expression of ectotherm animals has been studied mostly in fish [e.g. 36,37], but less in insects. Our results with ants agree with these studies, and show higher gene expression levels in the colder environment. In contrast, the direct influence of environmental humidity on the level of gene expression is rarely studied in general. Thus, our study provides novel insight into the reaction norms in gene expression to both environmental temperature and humidity on gene expression in an ant.

## Supporting information

S1 File S1_SupplementaryMaterial.pdf. (PDF)

S2 File S2_SurvivalData.txt. Survival data for the bioassay (formatted as individual survival for analysis with the survival package in R).
(TXT)

S3 File S3_GeneExpressionData.txt. Normalized Ct values of all candidate genes.
(TXT)
