# Supplementary material for: Survival and gene expression under different temperature and humidity regimes in ants
Source: PLoS One. 2017 Jul 31;12(7):e0181137. doi: 10.1371/journal.pone.0181137 (PMC5536355; doi:10.1371/journal.pone.0181137)

Survival and gene expression under different temperature and humidity regimes in ants

D. Stucki[1,3], D. Freitak[1,2,3] & L. Sundström[1,3]

1) Centre of Excellence in Biological Interactions / Department of Biosciences, University of Helsinki, POBox 65 (Viikinkaari 1), FI-00014 Helsinki, Finland

2) Centre of Excellence in Biological Interactions, University of Jyväskylä, FI-40014 Jyväskylä, Finland

3) Tvärminne Zoological Station, University of Helsinki, FI-10900 Hanko, Finland

Supplementary Tables & Figures

Table A: Primer information

Gene	Abbrev	Forward	Reverse	Efficiency
Arylphorin	*Aryl*	5'-ACT TAG AAC GCC TGT CCA AC-3'	5'-GAG ATT CCA TGT CCT CCA AG-3'	99.9
β-1-3-Glucan-binding protein	*B1,3g*	5'-ATC TTA CAG GAC TGC CTC CA-3'	5'-AAC GTG AAT AAG GCA ATT CG-3'	95.7
Heat shock protein 75	*HSP75*	5'-AAG ATA GGA CTG CCA ACG AA-3'	5'-GCA AGA GAT GCA GAA GGT CT-3'	91.28
Insulin Receptor 3	*IR3*	5'-GCG AGC GTT TTC TGT AGG TA-3'	5'-CCC AGG AGC TCG ATA ACA AC-3'	105
Pro-Phenoloxidase	*PPO*	5'-TCC AAC TGC TGT AGG CTG TA-3'	5'-ACC TTC ACA ATC TCG GTC AT-3'	96.4
Toll-receptor	*Toll*	5'-GCT CCT TCG TGA CCT AGT CG-3'	5'-GTG AAA GCG CCA TTA TCC AT-3'	102.4

The Primer sequences used for qPCR gene expression analysis. Abbrev. indicates the abbreviation used in the main text and the tables.

Table B: Pairwise survival

Compared factor	Fixed factors		β±SE	z
Population Island-Mainland	dry	cold	-1.05±0.55	-1.90
	dry	hot	0.25±0.48	0.53
	moist	cold	-1.06±0.53	-1.99
	moist	hot	-0.30±0.49	-0.62
Temperature cold-hot	Island	dry	-1.44±0.34	-4.26
	Island	moist	-0.83±0.34	-2.47
	Mainland	dry	-0.14±0.30	-0.48
	Mainland	moist	-0.08±0.28	-0.29
Humidity dry-moist	Island	cold	-0.15±0.41	-0.37
	Island	hot	0.46±0.25	1.86
	Mainland	cold	-0.16±0.30	-0.54
	Mainland	hot	-0.10±0.28	-0.35

Post-hoc pairwise contrasts on survival after ants from two populations (Island=Furuskär / Mainland=Prästkulla) were allowed to acclimatize for ten days to two temperatures (cold=20°C / hot=25°C) and two humidities (dry=50% / moist=75%). Each main factor was compared under all conditions and the differences are given as the parameter estimate β plus/minus standard error. All p-values were adjusted for multiple comparisons using FDR-correction.

Table C: Preliminary PCAs for component selection

	Pre-experiment						Experiment					
	PC1	PC2	PC3	PC4	PC5	PC6	PC1	PC2	PC3	PC4	PC5	PC6
Eigenvalue	2.79	1.27	0.97	0.44	0.35	0.18	3.09	1.02	0.68	0.59	0.35	0.26
Proportion of Variance	0.47	0.21	0.16	0.07	0.06	0.03	0.52	0.17	0.11	0.10	0.06	0.04
Cumulative Variance	0.47	0.68	0.84	0.91	0.97	1.00	0.52	0.69	0.80	0.90	0.96	1.00

Data for the preliminary PCA without rotation. Principal components with an Eigenvalue > 1.0 were retained for analysis.

Table D: Loadings of retained principal components

	Pre-experiment			Experiment	
	PC1	PC2	PC3	PC1	PC2
Aryl	-0.88	0.03	0.02	0.78	-0.14
B1,3g	0.85	0.11	0.34	0.37	0.71
HSP75	0.08	0.93	-0.06	0.77	0.20
IR3	-0.14	0.81	-0.03	0.85	-0.08
PPO	0.02	-0.08	0.98	0.52	0.36
Toll	0.82	-0.20	-0.29	-0.13	0.93

Loadings of each gene on the selected Principal Components from separate PCAs on pre-experiment gene expression and gene expression after ten days in the experiment. The principal components were rotated using oblique (oblimin) roataion.

Table E: Pairwise contrasts on PC scores reflecting gene expression

			PC1			PC2		
Compared factor	Fixed factors		β±SE	z	adj. p	β±SE	z	adj. p
Population Island-Mainland	dry	cold	1.63±0.15	10.86	<0.0001	1.49±0.18	8.09	<0.0001
	dry	hot	2.06±0.15	13.60	<0.0001	1.79±0.19	9.67	<0.0001
	moist	cold	1.73±0.15	11.57	<0.0001	1.83±0.18	9.95	<0.0001
	moist	hot	0.95±0.15	6.17	<0.0001	0.82±0.19	4.31	<0.0001
Temperature cold-hot	Island	dry	0.56±0.12	4.63	<0.0001	0.07±0.14	0.53	0.60
	Island	moist	-0.05±0.12	-0.45	0.66	-0.31±0.14	-2.22	0.0696
	Mainland	dry	0.45±0.14	3.24	0.0035	-0.27±0.16	-1.65	0.11
	Mainland	moist	1.05±0.14	7.26	<0.0001	0.66±0.17	3.92	0.0002
Humidity dry-moist	Island	cold	0.12±0.12	1.01	0.34	-0.25±0.14	-1.76	0.19
	Island	hot	-0.49±0.12	-4.04	0.0001	-0.63±0.14	-4.50	<0.0001
	Mainland	cold	-0.31±0.14	-2.19	0.0380	-0.55±0.16	-3.37	0.0026
	Mainland	hot	0.29±0.14	2.04	0.0492	0.38±0.17	2.27	0.0492

Post-hoc pairwise contrasts on the selected PC scores reflecting gene expression after ants from two populations (Island=Furuskär / Mainland=Prästkulla) were allowed to acclimatize for ten days to two temperatures (cold=20°C / hot=25°C) and two humidities (dry=50% / moist=75%). Each factor was compared under all conditions and the differences are given as the parameter estimate β plus/minus standard error. All p-values were adjusted for multiple comparisons using FDR-correction.

Table F: Pairwise contrasts on gene expression

Compared factor	Fixed factors		*Aryl* (storage/immune gene) β±SE	z	adj. p	*IR3* (insulin receptor) β±SE	z	adj. p	*HSP75* (heat-shock protein) β±SE	z	adj. p
Population Island-Mainland	dry	cold	-2.67±0.52	-5.15	<0.0001	-2.06±0.34	-6.1	<0.0001	-2.30±0.29	-7.95	<0.0001
	dry	hot	-3.64±0.52	-7	<0.0001	-2.70±0.34	-7.92	<0.0001	-2.99±0.29	-10.26	<0.0001
	moist	cold	-2.24±0.52	-4.33	<0.0001	-2.36±0.34	-6.98	<0.0001	-2.86±0.29	-9.89	<0.0001
	moist	hot	-1.08±0.53	-2.04	0.078	-1.28±0.35	-3.64	0.0012	-1.55±0.30	-5.2	<0.0001
Temperature cold-hot	Island	dry	-0.37±0.36	-1.05	0.39	-1.03±0.29	-3.6	0.0046	-0.67±0.23	-2.91	0.0189
	Island	moist	-0.11±0.36	-0.31	0.76	0.29±0.29	1	0.5201	0.26±0.23	1.12	0.5201
	Mainland	dry	-0.80±0.41	-1.94	0.11	-0.73±0.33	-2.21	0.0728	-0.11±0.27	-0.4	0.7532
	Mainland	moist	-2.67±0.43	-6.24	<0.0001	-1.14±0.34	-3.31	0.0028	-1.18±0.28	-4.27	0.0001
Humidity dry-moist	Island	cold	0.45±0.36	1.26	0.55	-0.67±0.29	-2.35	0.13	-0.05±0.23	-0.2	0.97
	Island	hot	0.71±0.36	1.98	0.078	0.64±0.29	2.24	0.0436	0.88±0.23	3.8	0.0009
	Mainland	cold	1.43±0.41	3.44	0.0041	-0.03±0.33	-0.09	0.93	0.65±0.27	2.42	0.047
	Mainland	hot	-0.45±0.42	-1.06	0.39	-0.44±0.34	-1.29	0.35	-0.43±0.27	-1.56	0.32

Compared factor	Fixed factors		*B1,3g* (immune gene) β±SE	z	adj. p	*PPO* (immune gene) β±SE	z	adj. p	*Toll* (immune gene) β±SE	z	adj. p
Population Island-Mainland	dry	cold	-2.50±0.23	-10.71	<0.0001	-2.70±0.42	-6.49	<0.0001	-1.81±0.31	-5.91	<0.0001
	dry	hot	-2.83±0.24	-12.04	<0.0001	-2.91±0.42	-6.92	<0.0001	-2.49±0.31	-8.05	<0.0001
	moist	cold	-2.40±0.23	-10.3	<0.0001	-3.38±0.42	-8.12	<0.0001	-2.50±0.31	-8.16	<0.0001
	moist	hot	-1.08±0.24	-4.48	<0.0001	-2.07±0.43	-4.79	<0.0001	-1.02±0.32	-3.21	0.0032
Temperature cold-hot	Island	dry	-0.37±0.18	-2.07	0.0693	-1.00±0.37	-2.72	0.0315	0.13±0.26	0.51	0.61
	Island	moist	0.38±0.18	2.16	0.1265	-0.26±0.37	-0.7	0.6324	0.55±0.26	2.11	0.1265
	Mainland	dry	-0.46±0.20	-2.25	0.0728	-0.32±0.42	-0.75	0.6324	0.82±0.30	2.76	0.0527
	Mainland	moist	-1.37±0.21	-6.42	<0.0001	-1.10±0.44	-2.49	0.0253	-0.93±0.31	-2.98	0.006
Humidity dry-moist	Island	cold	-0.01±0.18	-0.03	0.97	0.15±0.37	0.42	0.97	0.55±0.26	2.11	0.13
	Island	hot	0.74±0.18	4.18	0.0004	0.89±0.37	2.42	0.0403	0.96±0.26	3.7	0.001
	Mainland	cold	0.33±0.21	1.59	0.18	0.36±0.43	0.85	0.63	1.23±0.30	4.07	0.0008
	Mainland	hot	-0.58±0.21	-2.75	0.0611	-0.42±0.44	-0.96	0.51	-0.52±0.31	-1.69	0.30

Post-hoc pairwise contrasts on the expression of each gene after ants from two populations (Island=Furuskär / Mainland=Prästkulla) were allowed to acclimatize for ten days to two temperatures (cold=20°C / hot=25°C) and two humidities (dry=50% / moist=75%). Each factor was compared under all conditions and the differences are given as the parameter estimate β plus/minus standard error. All p-values were adjusted for multiple comparisons using FDR-correction.

Table G: Comparison of pre-experiment and condition-adapted gene expression

	Aryl (storage/immune gene)			*IR3* (insulin receptor)			*HSP75* (heat-shock protein)		
	β±SE	z	adj. p	β±SE	z	adj. p	β±SE	z	adj. p
I, cold, dry	0.52±0.36	1.47	0.43	0.05±0.28	0.19	0.85	0.71±0.22	3.18	0.0078
I, hot, dry	0.90±0.36	2.51	0.0392	1.08±0.28	3.91	0.0011	1.38±0.22	6.18	<0.0001
I, cold, moist	0.07±0.36	0.21	0.86	0.73±0.28	2.62	0.0261	0.76±0.22	3.39	0.0056
I, hot, moist	0.19±0.36	0.52	0.81	0.44±0.28	1.56	0.28	0.50±0.22	2.21	0.16
M, cold, dry	-1.32±0.41	-3.2	0.0162	-0.88±0.32	-2.76	0.0261	-0.09±0.26	-0.34	0.84
M, hot, dry	-0.52±0.41	-1.27	0.49	-0.15±0.32	-0.47	0.87	0.02±0.26	0.07	0.94
M, cold, moist	-2.77±0.42	-6.65	<0.0001	-0.85±0.32	-2.64	0.0174	-0.74±0.26	-2.85	0.0104
M, hot, moist	-0.07±0.43	-0.18	0.86	0.29±0.33	0.87	0.61	0.45±0.27	1.71	0.50
	B1,3g (immune gene)			*PPO* (immune gene)			*Toll* (immune gene)		
	β±SE	z	adj. p	β±SE	z	adj. p	β±SE	z	adj. p
I, cold, dry	0.74±0.18	4.07	0.0002	-0.29±0.35	-0.83	0.56	1.46±0.25	5.83	<0.0001
I, hot, dry	1.11±0.18	6.08	<0.0001	0.70±0.35	1.99	0.0941	1.33±0.25	5.3	<0.0001
I, cold, moist	0.75±0.18	4.11	0.0004	-0.45±0.35	-1.26	0.39	0.92±0.25	3.65	0.0016
I, hot, moist	0.37±0.18	1.98	0.16	-0.19±0.36	-0.53	0.71	0.37±0.25	1.46	0.28
M, cold, dry	-0.88±0.21	-4.21	0.0004	-1.23±0.41	-3.02	0.0204	-0.15±0.29	-0.52	0.73
M, hot, dry	-0.42±0.21	-2.01	0.14	-0.91±0.41	-2.24	0.14	-0.97±0.29	-3.37	0.0073
M, cold, moist	-1.20±0.21	-5.66	<0.0001	-1.60±0.41	-3.87	0.0013	-1.36±0.29	-4.65	<0.0001
M, hot, moist	0.15±0.22	0.69	0.61	-0.49±0.42	-1.16	0.61	-0.46±0.30	-1.53	0.50

Pairwise comparison of the gene expression level under each experimental condition with the pre-experiment level of gene expression (I=Island=Furuskär / M=Mainland=Prästkulla). Differences are given as the parameter estimate β plus/minus standard error. All p-values were adjusted for multiple comparisons using FDR-correction.

Figure A: Scree plots for principal component selection

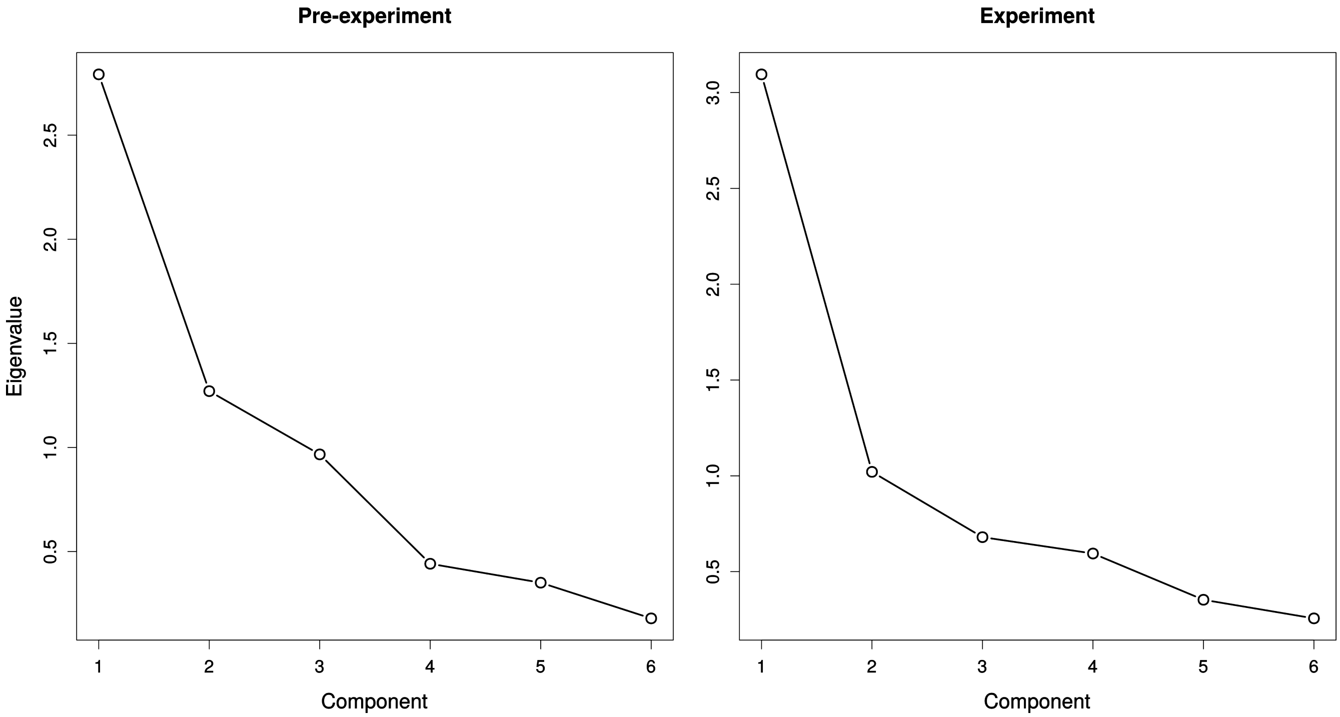

Supplement: S1 File — (PDF) [file pone.0181137.s001.pdf]
